# Potential role of sirtuin 1 in Müller glial cells in mice choroidal neovascularization

**DOI:** 10.1371/journal.pone.0183775

**Published:** 2017-09-08

**Authors:** Tomoka Ishida, Takeshi Yoshida, Kosei Shinohara, Kejia Cao, Ken-ichi Nakahama, Ikuo Morita, Kyoko Ohno-Matsui

**Affiliations:** 1 Department of Ophthalmology and Visual Science, Graduate School of Medical and Dental Sciences, Tokyo Medical and Dental University, Tokyo, Japan; 2 Department of Cellular Physiological Chemistry, Graduate School of Medical and Dental Sciences, Tokyo Medical and Dental University, Tokyo, Japan; 3 Department of Nanomedicine (DNP), Graduate School of Medical and Dental Sciences, Tokyo Medical and Dental University, Tokyo, Japan; University of Florida, UNITED STATES

## Abstract

This study investigated the potential role of sirtuin 1 in Müller glial cells in choroidal neovascularization. In the in vitro study, primary Müller glial cells were cultured and treated with resveratrol, a sirtuin 1 activator. Glial fibrillary acidic protein expression and angiogenesis-related gene expression were examined using quantitative polymerase chain reaction and phagocytosis, as a marker of Müller glial cell function; in addition, a latex bead assay was used to analyze cell function. For the *in vivo* study, choroidal neovascularization was induced in C57BL/6 mice via laser photocoagulation, and resveratrol was administered intravitreally. Eyecup whole mounts were created to measure choroidal neovascularization volumes on day 7. Immunohistochemical analysis with anti-glial fibrillary acidic protein antibody was used to detect Müller glial cell activation in eyes with choroidal neovascularization on day 1, 3, 5, and 7 after laser surgery. Resveratrol significantly promoted glial fibrillary acidic protein, anti-angiogenic factor, pigment epithelium-derived factor, and thrombospondin-1 expression in the cells as well as the phagocytic activities. Treatment of the choroidal neovascularization model with resveratrol resulted in early activation of Müller glial cells near choroidal neovascularization sites. Resveratrol-activated cells but not the controls migrated to the top of choroidal neovascularization sites and into the lesions from day 3. Resveratrol reduced the choroidal neovascularization size relative to controls. In conclusion, sirtuin 1 activation in Müller glial cells suppressed the development of choroidal neovascularization, and therefore, might be a therapeutic option.

## Introduction

Choroidal neovascularization (CNV) is the pathological growth of abnormal new blood vessels from the choroid into the sub-retinal space. CNV develops in certain conditions including age-related macular degeneration (AMD), pathologic myopia, angioid streaks, trauma, and inflammation [1]. In particular, AMD characterized by CNV is the leading cause of blindness among the elderly in developed countries [2]. Recently, anti-vascular endothelial growth factor (VEGF) drugs have been used for the treatment of CNV [3]. However, some patients show decreased responses to anti-VEGF drugs [4], and the consequent delay in CNV regression can lead to serious retinal damage resulting in irreversible and considerable vision loss [5]. The pathogenesis of CNV and the mechanism of its regression are not fully understood.

Müller glial cells (MGCs) are a specialized type of glia found only in the retina, spanning from the inner limiting membrane of the vitreous surface to the outer limiting membrane of the subretinal space [6]. MGCs play critical roles in maintaining retinal homeostasis and metabolism through the expression of neuroactive signaling molecules, and the phagocytosis of outer segment discs shed from photoreceptors [6]. Furthermore, MGCs express anti-angiogenetic factors such as pigment epithelium-derived factor (PEDF) and thrombospondin-1 (TSP-1) under normal conditions [6,7]. Increased expression of glial fibrillary acidic protein (GFAP), which indicates MGC activation, has been observed in patients with AMD [8] and murine CNV models [9]. This observation suggests that MGCs contribute to some aspects of AMD pathogenesis. However, the precise involvement of MGCs in AMD pathogenesis remains unclear.

It has been reported that Sirtuin 1 (Sirt1), an NAD+-dependent histone deacetylase (HDAC), regulates cell senescence, DNA damage repair, and apoptosis, and can control longevity in response to caloric restriction in numerous organisms including yeast, worms, flies, and possibly mammals [10]. In addition to its function as an HDAC, Nagineni et al. [11] showed that resveratrol (RSV), a Sirt 1 activator, inhibited hypoxia-induced VEGF secretion in cultured human retinal pigment epithelial cells (RPE). Furthermore, Zhang et al. [12] showed that RSV downregulated VEGF receptor 2 phosphorylation in endothelial cells and Nagai et al. [13] reported that RSV administration prevented the development of laser-induced CNV in mice. However, the mechanisms by which RSV regulates angiogenesis are not fully understood.

Thus, to elucidate the mechanisms underlying Sirt1 activation-triggered early CNV regression, we administered RSV into the vitreous of a laser-induced CNV mice model and investigated the correlation between MGC activation and CNV regression.

## Materials and methods

### Cell culture

MGCs were isolated from murine pup eyes (post-natal day 4–8) as described previously [14–16], and were cultured in Dulbecco's modified Eagle's medium (DMEM, Sigma-Aldrich, St. Louis, MO, USA) containing 100 U/mL penicillin, 100 μg/mL streptomycin (Wako Pure Chemical Industries, Osaka, Japan), and 10% fetal bovine serum (FBS, Biowest, Nuaillé, France) in a 37°C humidified atmosphere containing 5% CO_2_. All experiments were performed using first to third passage cells.

### Sirt1 activity measurement

MGCs were seeded in six-wells plates and cultured to 100% confluency. RSV was diluted DMEM with 0.2% Dimethyl sulfoxide (DMSO, Sigma-Aldrich). And the vehicle was used as controls in following vitro study. The cells were incubated with or without 140 μM RSV for 12 h, and washed twice with PBS, and the protein was finally extracted. We measured the Sirt1 activity of the lysate using the Sirt1 fluorimetric drug discovery kit (ENZO Life Sciences International, Inc., Farmingdale, NY, USA) [17]. Samples were incubated with Sirt1 deacetylase substrate, 25μM) and NAD+ (500μM) in assay buffer at 37°C on a shaker for 45 min and then 2mM nicotinamide and developer were added followed by incubation at 37°C on the shaker for 10 min. The fluorescence was read using a plate-reading fluorimeter (excitation and emission wavelengths: 360 and 450 nm, respectively).

### Immunocytochemistry

MGCs were seeded on two-well glass slides (Nunc International, New York, NY, USA) and cultured to 100% confluency. The cells were then incubated with 140 μM RSV without FBS for 24 h, washed twice with PBS, fixed with 4% PFA, washed again, blocked using 10% normal goat serum, and incubated with anti-GFAP antibody overnight at 4°C. After three washes with PBS, the cells were incubated with fluorophore-conjugated secondary antibody for 1 h in the dark at 25°C, washed, and then mounted using mounting medium with 4',6-diamidino-2-phenylindole (DAPI, Vector Laboratories, Inc., Burlingame, CA, USA). Images were acquired using a laser scanning confocal microscope. GFAP- and DAPI-stained areas were measured using the ImageJ software (National Institutes of Health, NIH, Bethesda, MD, USA) [18].

### Light cycler real-time polymerase chain reaction (PCR)

MGCs were seeded in six-well plates until they were 100% confluent and then incubated in FBS-free DMEM overnight, following by incubation with or without 140 μM RSV for 6 h. Total RNA was then extracted from cultured MGCs using an illustra RNAspin Mini RNA Isolation kit (GE Healthcare, Buckinghamshire, UK). cDNA was synthesized from 1 μg total RNA using the ReverTra Ace (MMLV reverse transcriptase RNaseH-, TOYOBO, Osaka, Japan) according to the manufacturer’s instructions. Semi-quantitative polymerase chain reaction (PCR) was performed using KAPA SYBER FAST qPCR Master Mix (KAPA BIOSYSTEMS, Boston, MA, USA) using a LightCycler 480 II system (Loche, Mannheim, Germany) to detect the expression of PEDF, TSP-1. We used the geometric mean values of three housekeeping genes (β-actin, GAPDH, and Ywhaz) as a normalization factor. The amplification schedule was as follows: initial denaturation at 95°C, followed by 40–45 cycles of 95°C for 30 s, 62°C (PEDF, TSP-1, and GAPDH), or 55°C (β-actin) or 64°C (Ywhaz) for 1 min, and 72°C for 30 s. The following primer sequences were used: PEDF forward, 5’-cacccgacttcagcaagattact-3’ and reverse, 5’-tcgaaagcagccctgtgtt-3’ (GenBank accession number: NC 000077.6) TSP-1 forward, 5’-aacaaaggctgctccagctc-3’ and reverse 5’-ggatgctgcctgcagagtg-3’ (GenBank accession number: NC 000068.7), GAPDH forward, 5’- TGACCACAGTCCATGCCATC-3’ and reverse, 5’-ACTTGGCAGGTTTCTCCAGG-3’ (GenBank accession number: NC_000072.6), and Ywhaz forward, 5’-cgaggttgctgctggtgat-3’ and reverse, 5’gtcggctgcatctccttttt-3’ (GenBank accession number: NC_000081.6). The β-actin primers were commercially purchased (Qiagen, Hilden, Germany). The relative change in mRNA expression was calculated using ^ΔΔ^CT values, and each experiment was performed in triplicate. Levels were normalized to those of β-actin and reported as fold change compared to the controls.

### In vitro phagocytosis assays

MGCs were seeded onto 35-mm dishes (Ibidi GmbH Martinsried, Germany) and cultured to 30% confluence. Then, the cells were incubated with fluorescent latex beads (diameter: 2.0-μm, 2.5% aqueous suspension, 1:2000, Sigma-Aldrich, St. Louis, MO, USA) with or without 140 μM RSV for 24 h, after which they were prepared and observed as described previously for the immunocytochemistry. GFAP and bead signal areas were analyzed using the ImageJ software.

### Animals

All animal study protocols were approved by the Animal Care and Use Committee of the Tokyo Medical and Dental University (Permit Number: 0150345A, 0160055A, 0160408A, and 0170172A). Experiments were performed using C57BL/6JJcl mice (CLEA Japan, Tokyo, Japan) in accordance with the Tokyo Medical and Dental University’s Guidelines for the Care and Use of Animals. The mice were housed under standard conditions of humidity, temperature, and dark/light cycle. They were fed a laboratory rodent diet and tap water. All treatments were performed under anesthesia, and all attempts were made to minimize animal suffering. The humane endpoint criteria were determined to be when the animals exhibited respiratory disturbance, abnormal behaviors, or difficulty in consuming water and food. During this experiment, we used totally 64 mice. Among them, eight mice died during the laser coagulation and intravitreous injection because of vagus nerve reflex, and one mouse died the day after the medical procedures. We observed the animals for 2 h after the procedures and every day until euthanasia. Finally, 55 mice were euthanized at specified time points for this study.

### Laser-induced CNV

CNV was induced in mice using a modified laser-induced CNV method [19,20]. Mice (6-week-old) were anesthetized using 40 mg/kg pentobarbital (Kyouritu Seiyaku Corporation, Tokyo, Japan) for all procedures. The pupils were dilated with 0.5% phenylephrine hydrochloride and 0.5% tropicamide (Santen Pharmaceutical Co., Ltd., Tokyo, Japan), and four photocoagulation lesions were induced per fundus using a diode green laser (150 mW, 0.05 s, 75 μm) between the retinal vessels in a peripapillary distribution using a slit-lamp delivery system (Ultima 2000SE, Coherent, Santa Clara, CA, USA) with a handheld coverslip as a contact lens. The formation of a subretinal bubble during laser treatment confirmed the rupture of Bruch’s membrane.

### Intravitreal injection of RSV

Mice were administered 1 μL of 30 μM RSV (Tokyo Chemical Industry Co., LTD, Tokyo, Japan) diluted in PBS into the right eye (Gibco, Palo Alto, CA) or 1 μL of PBS as control into the left eye with a 30-gauge needle introduced into the vitreous at 200 μm posterior to the limbus on the same day as the laser surgery. To minimize injection outflow and prevent infection, ofloxacin (Santen Pharmaceutical Co.) was applied to the injection site and surrounding external areas. Caution was exercised to avoid damaging the retina and lens.

### Retinal flat mounting

Eyes from laser-induced CNV mice (n = 10) were enucleated on day 7 after laser treatment, and the cornea, lens, and retina were removed. Dissected eyecups were fixed with 4% paraformaldehyde overnight and washed with PBS buffer containing 0.5% Triton-X. After blocking with 1% bovine serum albumin in PBS/Triton-X for 1 h, the endothelial cell marker fluorescein-conjugated isolectin B4 (1:200, Vector Laboratories, Inc., Burlingame, CA, USA) was added and incubated at 4°C overnight. The eyecups were washed with PBS/Triton-X and placed on slides, mounted with Mount-Quick “Aqueous” (Daido Sangyo Co., Ltd., Saitama, Japan), and imaged using a laser scanning confocal microscope (LSM, model 510; Carl Zeiss, Oberkochen, Germany).

### CNV volume quantification

Z-stack images of CNV retinal flatmounts stained with isolectin B4 were acquired using a laser scanning confocal microscope with a 10× objective lens. The image stacks were rendered in 3D using the IMARIS imaging software (Carl Zeiss) and processed to digitally extract the fluorescent lesion volume.

### Immunohistochemical analysis of GFAP

Mice were euthanized for immunohistochemical analysis on day 1, 3, 5, and 7 after laser surgery and intravitreal injections (n = 4 per time point). The mouse eyes were enucleated and fixed with 1% paraformaldehyde for 30 min at 25°C, after which the cornea and lens were removed and fixed again with 1% paraformaldehyde for 30 min at 25°C and washed with PBS. The eyecups were perfused with 0.1 M phosphate-buffered 10% sucrose for 1 h at 25°C, and 0.1 M phosphate buffered 20% sucrose overnight at 4°C, then frozen in optimal cutting temperature (OCT) compound (Sakura Finetek Japan, Tokyo, Japan) using liquid nitrogen. The frozen blocks were cut into 10-μm sections for immunohistochemical analysis, and were washed thrice with PBS and blocked with 10% normal goat serum in PBS. The sections were then incubated with a polyclonal rabbit anti-GFAP primary antibody (DACO Japan, Kyoto, Japan) (IR524, antigen, GFAP isolated from cow spinal cord) [9] overnight at 4°C, washed with PBS, and then incubated with appropriate fluorophore-conjugated secondary antibodies for 1 h in the dark at 25°C. After washing three times with PBS, the sections were mounted with Mount-Quick “Aqueous” (Daido Sangyo Co., Ltd.) and imaged using a laser scanning confocal microscope.

### Statistics

Each experiment was performed at least three times, and all the data were analyzed using the Student’s *t*-test. A p-value < 0.05 was considered statistically significant, and the data are expressed as the mean ± standard error of the mean (SEM) or standard deviation (SD).

## Results

### Activation of Sirt 1 by RSV activated cultured MGCs

**Fig 1:**
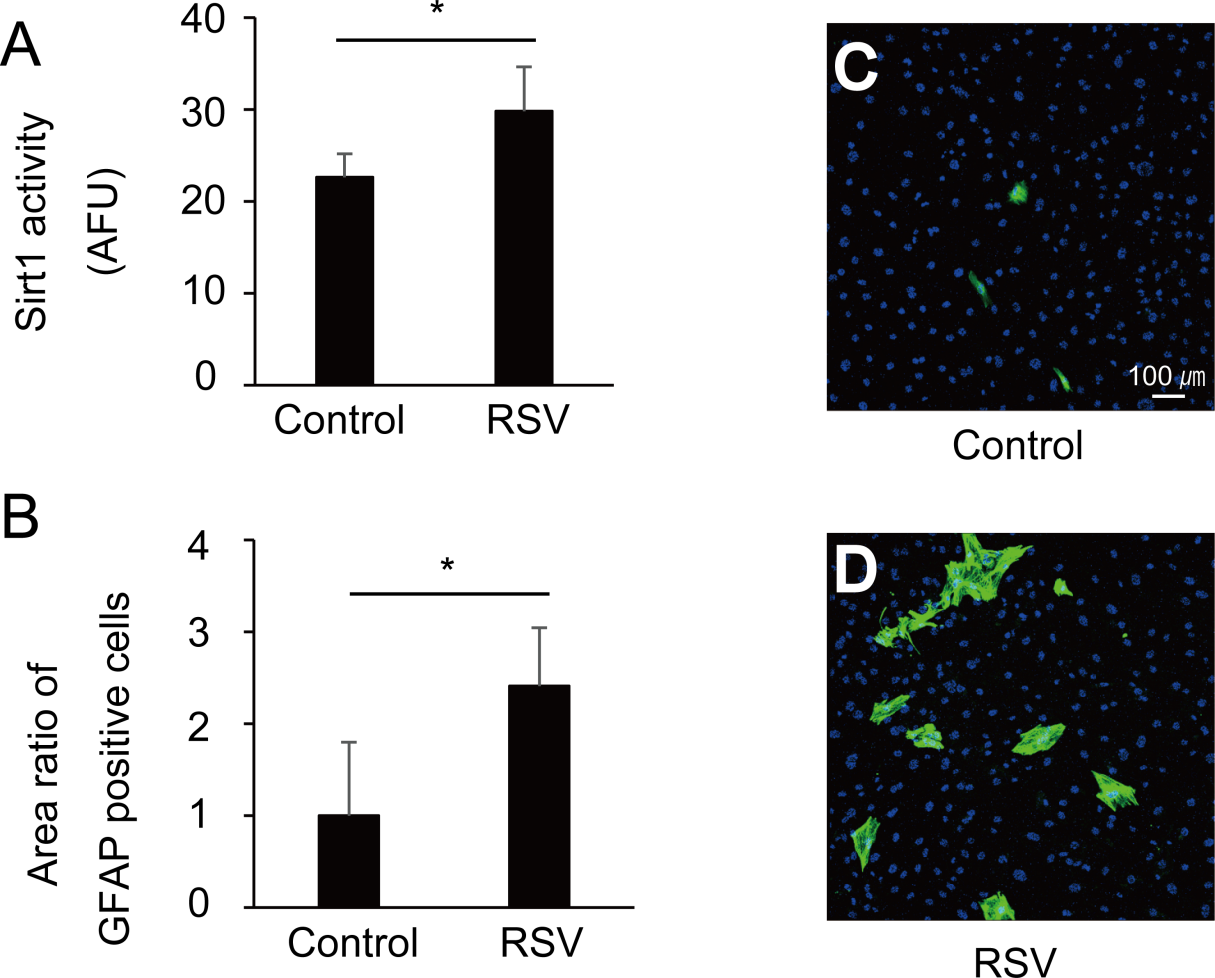
Resveratrol (RSV) treatment activated cultured Müller glial cells MGCs). (A) The activity of Sirt1 in MGCs treated with RSV. Sirt1 activity is expressed as arbitrary fluorescence units (AFU). RSV increased the Sirt1 activity by 1.3-fold relative to control (p < 0.01). (B) The ratio of glial fibrillary acid protein (GFAP)-positive and 4',6-diamidino-2-phenylindole (DAPI)-labeled cell nuclear areas (mean ratio ± SD). RSV treatment significantly increased GFAP-positive MGCs by 2.4-fold relative to control (p < 0.01). (C and D) Representative images of untreated and RSV-treated MGCs labeled with anti-GFAP antibody (green) and DAPI (blue, ×100).

We studied the change of activity of Sirt1 in MGCs treated with RSV. RSV significantly increased the activity of Sirt1 by up to 1.3-fold comparing with the control (Fig 1A, p < 0.01). Further, RSV treatment significantly increased the number of GFAP-positive MGCs by up to 2.4-fold relative to that of the control (Fig 1B–1D, p < 0.01).

### Activation of Sirt 1 in MGC involved gene expression and functions related to CNV regression

**Fig 2:**
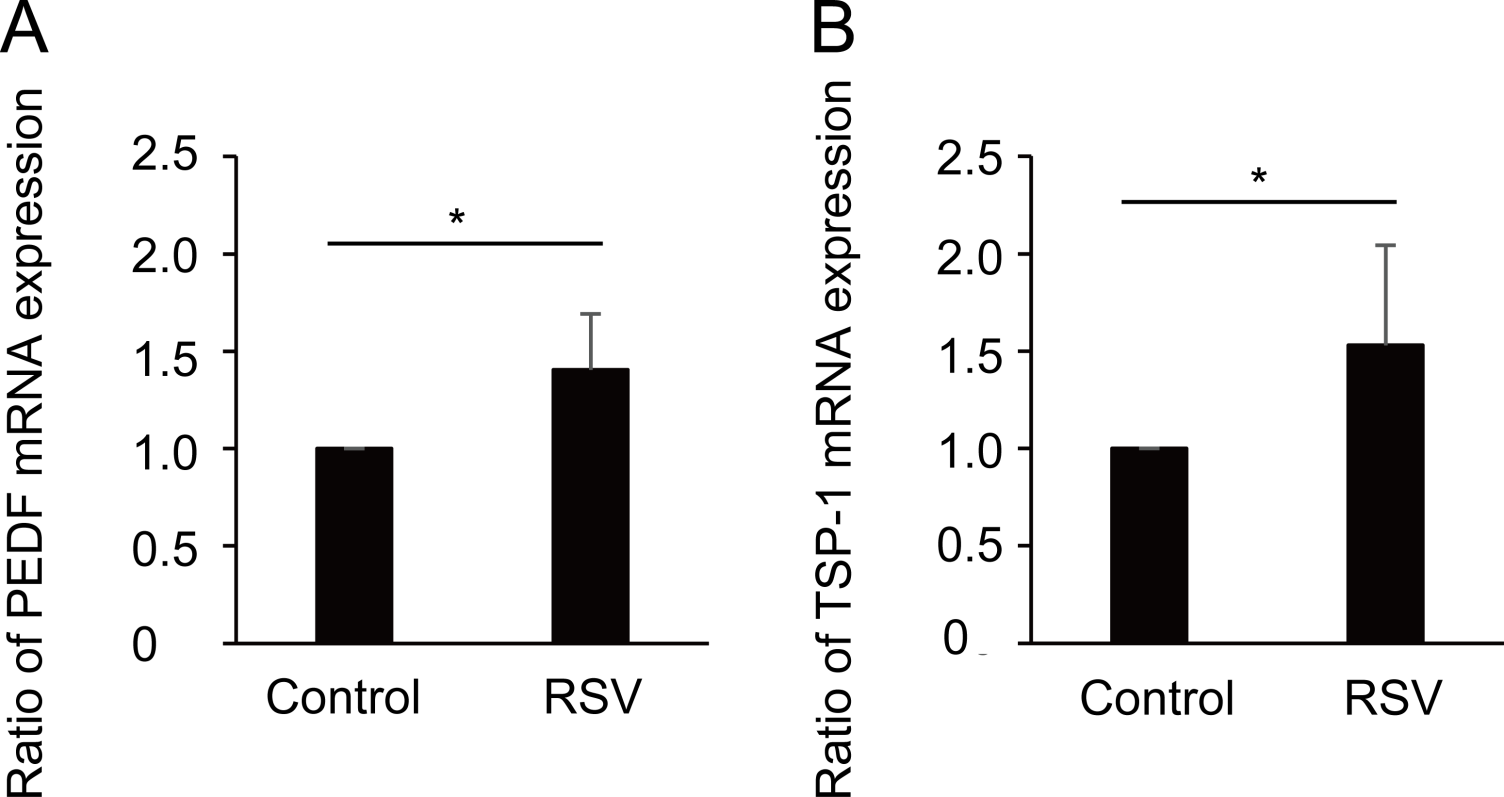
Resveratrol (RSV) promoted pigment epithelium-derived factor (PEDF) and thrombospondin-1 (TSP-1) mRNA expression in Müller glial cells (MGCs). (A and B) RSV treatment significantly increased PEDF and TSP-1 mRNA expression by 1.4 and 1.5-fold, respectively relative to control (p < 0.05).

Next, we focused on the involvement of Sirt 1 in the expression of CNV regression-related genes and determined whether Sirt1 activation in MGC enhanced anti-angiogenic factors. RSV treatment increased the gene expression of anti-angiogenic factors, PEDF and TSP-1, by 1.4- and 1.5-fold, respectively (Fig 2A and 2B, p < 0.05).

**Fig 3:**
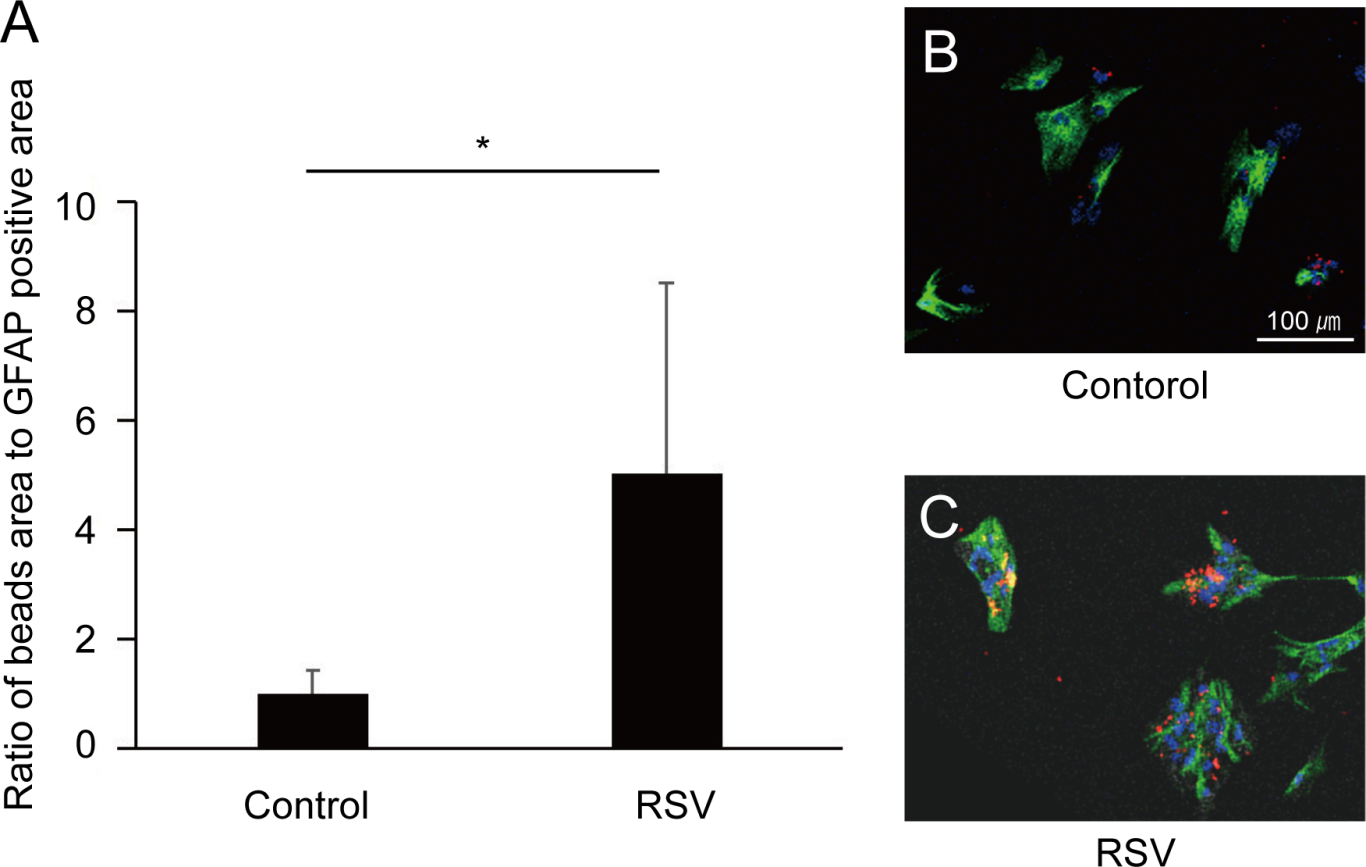
Phagocytic ability of cultured Müller glial cells (MGCs). (A) The ratio of glial fibrillary acid protein (GFAP)-positive area to bead-signal area (mean ratio ± standard deviation, SD). Resveratrol (RSV) treatment significantly increased bead phagocytosis by up to 5.0-fold relative to control (p < 0.05). (B and C) MGCs cultured with latex beads (red) are shown (×100). Representative images of untreated and RSV-treated cells labeled with anti-GFAP antibody (green) and 4',6-diamidino-2-phenylindole (DAPI, blue).

We next examined the phagocytic activity in activated MGCs by RSV. RSV treatment increased MGCs phagocytosis of latex beads by 5.0-fold compared to the non-treated control cells (Fig 3A–3C, p < 0.05).

### RSV regulated experimental CNV development

**Fig 4:**
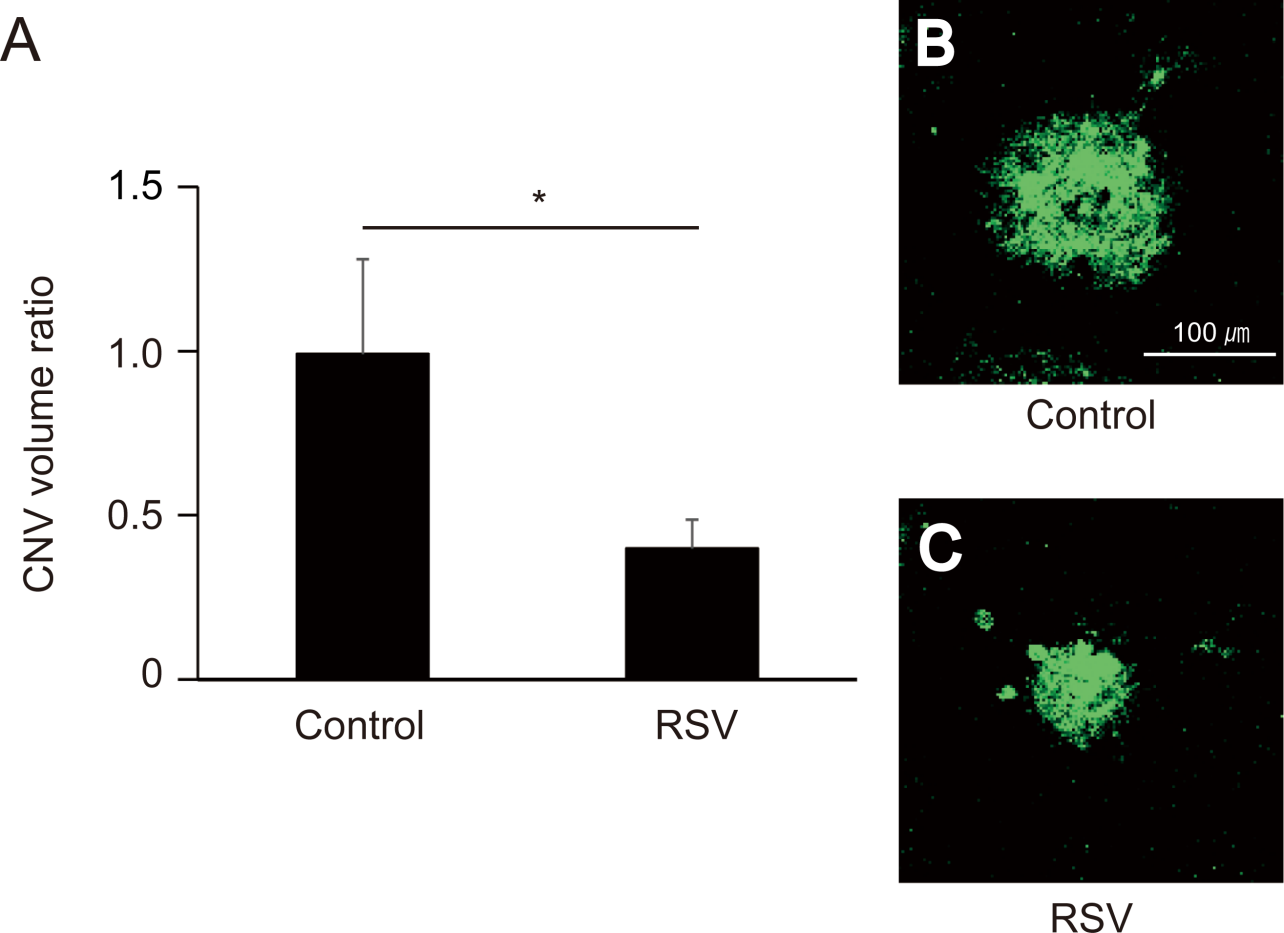
Laser-induced choroidal neovascularization (CNV) volume at 7 days post laser application and intravitreal RSV injection. (A) CNV volume (mean ± standard error of the mean, SEM). RSV administration (30 μM) significantly suppressed CNV volume relative to control (p < 0.05). (B and C) CNVs from untreated and RSV-treated animals labeled with isolectin B4 (×100).

We next examined RSV anti-angiogenic effect in vivo using experimental CNV murine model. The extent of laser-induced CNV in murine eyes was measured on day 7 after laser application. In this model, RSV administration (30 μM) significantly suppressed the CNV volume to 40.0% of control levels (Fig 4A–4C, p < 0.05).

### RSV promoted MGC migration to CNV lesions

**Fig 5:**
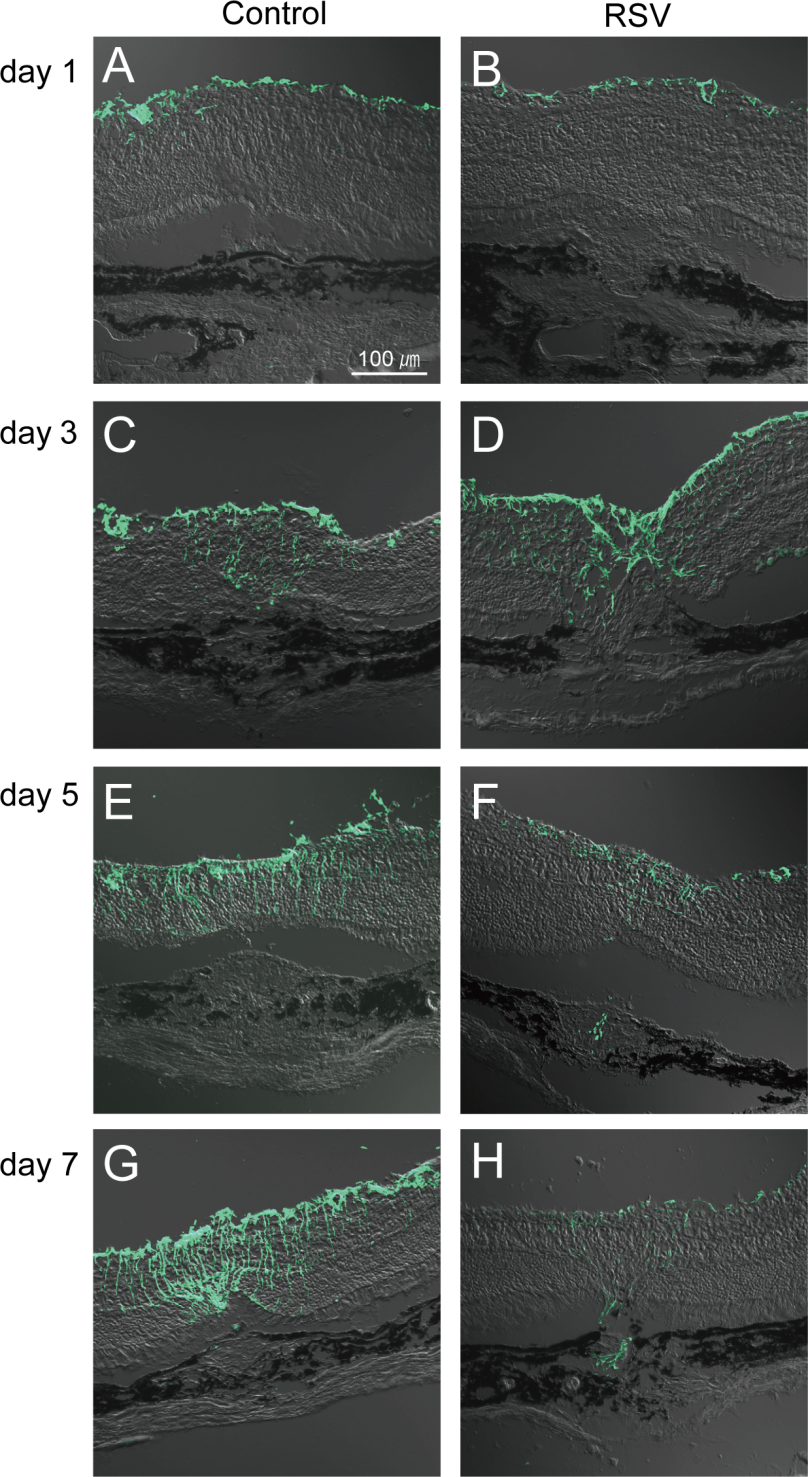
Glial fibrillary acid protein (GFAP)-positive Müller glial cells (MCGs) in resveratrol (RSV)-treated choroidal neovascularization (CNV)-induced eyes. Activated MGCs were labeled with anti-GFAP antibody (×200). (A and B) On day 1, GFAP-positive MGCs were not present. (C and D) On day 3, GFAP-positive MGCs were present in both RSV-treated and control animals, but cells only migrated to the CNV lesion surface and into the CNV in treated eyes. (E and F) On day 5, RSV-treated animals presented both reduced MGC activity and CNV size, whereas control animals showed increases in both. Again, MGCs were found inside CNV lesions in RSV-treated animals but not controls. (G and H) On day 7, MGC numbers and CNV size continued to decrease in RSV-treated eyes relative to controls. GFAP-positive MGCs were also observed in CNV lesions only in the RSV-treated eyes.

We studied the correlation of MGC activation and MGCs migration in retina around laser-induced CNV in murine eyes. On day 1, no GFAP-positive MGCs were observed in both the control and RSV-treated animals (Fig 5A and 5B). On day 3, GFAP-positive MGCs were observed in both the control and RSV-treated animals, but the latter showed much higher quantities in the fields (Fig 5C and 5D). Particularly, GFAP-positive MGCs in RSV-treated animals had migrated to the surface of CNV lesions missing the RPE layer, and some were present in the CNV lesions (Fig 5D). On day 5, CNV sizes in the RSV-treated animals were smaller than those in the controls were, and the retinal GFAP-positive MGC count decreased relative to that obtained on day 3. However, the control mice showed an increase in the GFAP-positive MGC count (Fig 5E and 5F). GFAP-positive MGCs were still present in CNV lesions in the RSV-treated eyes (Fig 5F), but not in the controls (Fig 5E). Finally, on day 7, the CNV size of the RSV-treated eyes continued to decrease relative to that reported for the controls (Fig 5G and 5H). GFAP-positive MGCs in RSV-treated retinas decreased to the point of almost disappearing (Fig 5H), whereas they continued to increase in control retinae (Fig 5G). Similarly, GFAP-positive MGCs were still found inside CNV lesions in RSV-treated eyes (Fig 5H), but not in the controls (Fig 5G).

## Discussion

In the present study, we first demonstrated that RSV-activated MGCs showed increased expression of the antiangiogenic factors, PEDF and TSP-1, as well as the phagocytic ability. PEDF is well known to reduce migration and promote apoptosis of endothelial cells [21], and TSP-1 induces apoptosis of endothelial cells and prevents them from responding to a wide variety of angiogenic stimulators [22]. While MCGs are known to express PEDF and TSP-1 under normal conditions [6], we demonstrated that RSV treatment upregulated PEDF and TSP-1 expression, and may have anti-CNV properties. Interestingly, a previous study showed that intravitreal injection of 2-methoxyestradiol in retinopathy in a premature rat model demonstrated positive GFAP expression in MGCs and negative VEGF immunoreaction in retina [23]. This study indicates that MGCs activation strongly correlated with anti-angiogenic effect and enhances our results.

We also showed that RSV treatment induced phagocytosis of MGCs. It has been reported that CNS astrocytes actively phagocytized debris, including dead cells, in an in vitro model of brain injury [24]. It has also been suggested that phagocytosis protects healthy neurons from bystander cell death [24]. MGCs have natural phagocytic capabilities for maintaining retinal homeostasis, and it has been reported that MGCs phagocytize outer-segment discs shed under normal conditions [6] as well as bacteria and RPE layer debris [25,26]. Therefore, activated MGCs may phagocytize dead cell debris, neutralizing their toxicity and, thereby, regressing CNV.

Based on the in vitro results, we examined the animal CNV model to determine whether RSV administration affected CNV. The result revealed that RSV administration significantly suppressed CNV volume to 40.0% of control levels.

To the best of our knowledge, this is the first study to demonstrate the anti-angiogenic properties of RSV in MGCs using a laser-induced murine CNV model. Furthermore, our results strongly indicate that MGCs were associated with CNV development. In previous studies, RSV has been shown to suppress VEGF expression by inducing oxidative stress in RPE cells, resulting in an anti-angiogenic effect on CNV [11,12]. Furthermore, it has also been reported that RSV facilitated macrophage deactivation and prevented the proliferation and migration of endothelial cells [13,27]. In addition to these previous results, RSV activated MGCs, which were likely involved in the CNV regression.

We previously reported that differentiated RPE cells expressed high levels of both VEGF and PEDF, and a critical balance between these two was important to prevent CNV development and AMD [28,29]. Recently, anti-VEGF therapy has become a standard treatment for CNV [30], and while it is useful for stabilizing visual acuity [31] the actual recovery of visual acuity has proven difficult and non-responsive cases have been reported [4]. Therefore, it is possible that cells other than RPE are involved in CNV development. MGCs are known to have critical roles in maintaining retinal structural and functional homeostasis [6]. In contrast, MGCs have been observed in human AMD-affected retinas [8] and murine laser-induced CNV lesions [9].

It is interesting to note that the maximum number of activated MGCs was achieved on day 3 by the administration of RSV, but those in the control group still increased on day 7. In addition, activated MGCs migrated towards the surface of the CNV lesions that lacked the RPE layer, and RSV-activated MGCs were present inside the CNV lesions. Both of these phenomena were more remarkably pronounced in RSV-treated eyes than in the controls. Thus, RSV treatment promoted CNV regression by an RSV-induced activation of MGCs. The present study showed that activated MGCs migrated to the CNV surfaces missing the RPE layer. Previously, Lassota [32] similarly showed that MGCs formed a plaque on CNV surfaces where the RPE layer was missing in an animal CNV model, but could not determine the role of this particular plaque. During central nervous system (CNS) damage, it is known that brain astrocytes elongate and surround injured areas to restrict the spread of inflammatory cells to healthy tissues [33] These phenomena may be similar to what has been observed with MGCs and CNV lesions. Based on our findings, RSV treatment promoted the early formation of MGC plaques on the surfaces of CNV lesions, possibly restricting the spread of inflammatory and endothelial cell invasion from the CNV lesion, thereby protecting the healthy retina. This quarantine process may be key to accelerating CNV regression and maintenance of visual acuity.

In addition, a significant number of activated MGCs were found inside the CNV lesions in RSV-treated animals, but very few were found in the controls. This phenomenon was confirmed in a previous study, but the mechanism underlying its development remains unclear [9] Generally, cell motility is correlated with f-actin formation in cell bodies [34], and it has been reported that RSV promotes f-actin formation through deacetylation of the actin-binding protein cortactin in podocytes [35]. Indeed, RSV is an activator of Sirt 1, which deacetylates numerous sites of many proteins [36–38]. Therefore, RSV may promote the formation of f-actin in MGCs, inducing MGC migration. In the present study, RSV clearly promoted MGC migration into the CNV lesions, but the role of these cells in this location remained unclear. To clarify this, we investigated MGC expression of anti-angiogenic factors and phagocytic activity.

Recently, several studies have demonstrated that oral or intraperitoneal administration of RSV suppressed CNV by approximately 30% and 12%, respectively [13,27,39]. We found that intravitreal administration of RSV reduced the CNV volume by ~60% relative to the control, indicating that intravitreal RSV injection was more effective than the previously investigated routes. This observation is reasonable considering that an intravitreal injection can maintain high vitreous drug concentrations and presents a direct route to the retina.

In summary, RSV activated the MGCs, which then migrated to the surface of the CNV and inside the lesions. Activated MGCs distinguish CNV lesions from the healthy retina, and facilitate CNV regression, resulting in decreased retinal damage. MGCs have numerous functions in the maintenance of retinal homeostasis, and MGC activation may have a critical role in promoting early CNV regression. The present study is the first to focus on the relationship between RSV and MGCs in CNV treatment, and is the first step to developing a novel therapy for all patients with CNV. Further investigation is required to develop new treatments for CNV.
